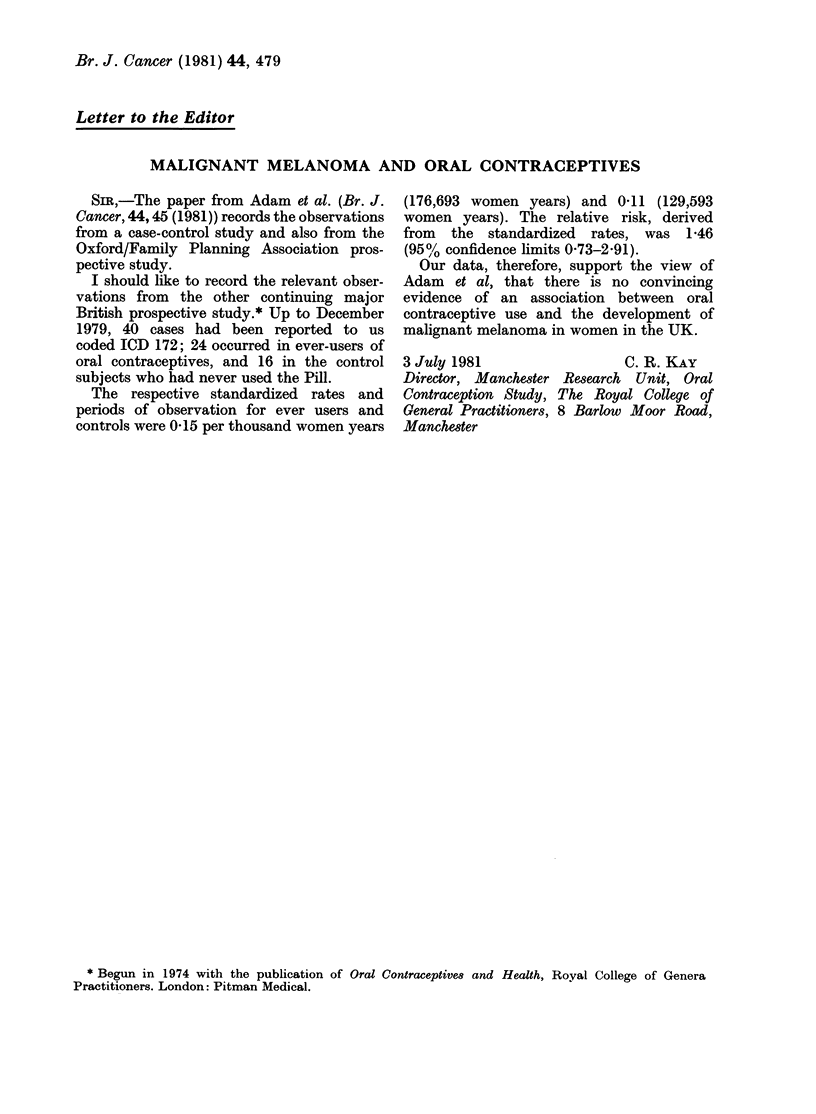# Malignant melanoma and oral contraceptives

**Published:** 1981-09

**Authors:** C. R. Kay


*Br. J. Cancer* (1981) **44**, 479

### Letter to the Editor

### MALIGNANT MELANOMA AND ORAL CONTRACEPTIVES

SIR,—The paper from Adam *et al.* (*Br. J. Cancer*, **44,** 45 (1981)) records the observations from a case-control study and also from the Oxford/Family Planning Association prospective study.

I should like to record the relevant observations from the other continuing major British prospective study.* Up to December 1979, 40 cases had been reported to us coded ICD 172; 24 occurred in ever-users of oral contraceptives, and 16 in the control subjects who had never used the Pill.

The respective standardized rates and periods of observation for ever users and controls were 0·15 per thousand women years (176,693 women years) and 0·11 (129,593 women years). The relative risk, derived from the standardized rates, was 1·46 (95% confidence limits 0·73–2·91).

Our data, therefore, support the view of Adam *et al*, that there is no convincing evidence of an association between oral contraceptive use and the development of malignant melanoma in women in the UK.

3 *July* 1981

C. R. KAY
*Director, Manchester Research Unit, Oral Contraception Study, The Royal College of General Practitioners,* 8 *Barlow Moor Road, Manchester*

* Begun in 1974 with the publication of *Oral Contraceptives and Health,* Royal College of Genera Practitioners. London: Pitman Medical.